# Enhancing remediation potential of heavy metal contaminated soils through synergistic application of microbial inoculants and legumes

**DOI:** 10.3389/fmicb.2023.1272591

**Published:** 2023-09-29

**Authors:** Kang Zheng, Zexun Liu, Chao Liu, Jiayi Liu, Jiayao Zhuang

**Affiliations:** Collaborative Innovation Center of Sustainable Forestry in Southern China of Jiangsu Province, Nanjing Forestry University, Nanjing, China

**Keywords:** co-remediation, soil contamination, high-throughput sequencing, bacterial community structure, legume

## Abstract

Soil microorganisms play a crucial role in remediating contaminated soils in modern ecosystems. However, the potential of combining microorganisms with legumes to enhance the remediation of heavy metal-contaminated soils remains unexplored. To investigate this, we isolated and purified a highly efficient cadmium and lead-tolerant strain. Through soil-cultivated pot experiments with two leguminous plants (*Robinia pseudoacacia* L. and *Sophora xanthantha*), we studied the effects of applying this microbial agent on plant nutrient uptake of soil nutrients, heavy metal accumulation, and the dynamics of heavy metal content. Additionally, we examined the response characteristics of inter-root microbial and bacterial communities. The results demonstrated that microorganisms screened from heavy metal-contaminated soil environments exhibited strong survival and adaptability in heavy metal solutions. The use of the *Serratia marcescens* WZ14 strain-phytoremediation significantly increased the soil’s ammonium nitrogen (AN) and organic carbon (OC) contents compared to monoculture. In addition, the lead (Pb) and cadmium (Cd) contents of the soil significantly decreased after combined remediation than those of the soil before potting. However, the remediation effects on Pb- and Cd-contaminated soils differed between the two legumes following the *Serratia marcescens* WZ14 inoculation. The combined restoration altered the composition of the plant inter-rhizosphere bacterial community, with the increase in the relative abundance of both Proteobacteria and Firmicutes. Overall, the combined remediation using the tolerant strain WZ14 with legumes proved advantageous. It effectively reduced the heavy metal content of the soil, minimized the risk of heavy metal migration, and enhanced heavy metal uptake, accumulation, and translocation in the legumes of *S. xanthantha* and *R. pseudoacacia*. Additionally, it improved the adaptability and resistance of both legumes, leading to an overall improvement in the soil’s environmental quality. These studies can offer primary data and technical support for remediating and treating Cd and Pb in soils, as well as rehabilitating mining sites.

## 1. Introduction

The issue of heavy metal pollution in soil, driven by unnatural factors from rapid industrialization, is increasingly severe and requires urgent solutions. Soil, as the most abundant and diverse ecosystem on Earth, plays a crucial role in reflecting soil health and function through the dynamics of soil quality, surface vegetation, and microbial communities in complex environments (Jiang et al., 2016). Mine remediation using microorganisms has been vastly studied over recent decade for remediation and ecological systems restoration at various mine sites (Xiao et al., 2023).

The unnatural uptake of heavy metals during mining is the primary cause of soil heavy metal contamination (Rachelle et al., 2018; Wang et al., 2022). Heavy metals (HMs) contamination has led to severe environmental issues, including soil nutrient losses, sharp reductions in soil microbial diversity, and hindered plant growths (Liu et al., 2019; Gonalves et al., 2020; Shuaib et al., 2021). Unlike organic pollutants, heavy metal pollution is characterized by difficult degradation, hidden, long-term, and high toxicity, and it is difficult to achieve the intended effect of complete removal in the short term in the remediation of soil heavy metal pollution (Luo et al., 2019). As traditional remediation techniques like physical and chemical methods are increasingly limited in addressing soil heavy metal pollution, phytoremediation has gained significant attention as an alternative due to its ecological, economic, and sustainable advantages (Fatima et al., 2016; Saxena et al., 2019; Xiao L. et al., 2021), emerging as one of the most promising remediation approaches. In addition, plants play a vital role in improving soil quality and optimizing the soil microbial community (Schloter et al., 2018; Beiyuan et al., 2021). Various microorganisms living in the rhizosphere, can have beneficial effects on plant growth, and health and increase plant biomass production (Evlat et al., 2023). Therefore, investigating changes in soil nutrients and microbial community structure during phytoremediation is crucial for successful ecological restoration.

Most studies on phytoremediation for soil heavy metal remediation, especially on phytoextraction, have primarily focused on utilizing super-enriched plants (HMH) to extract soil heavy metals (Duan et al., 2020; Atikur-Rahman et al., 2022). Although HMH has demonstrated favorable remediation results, certain studies have presented its limitations, such as slow growth and shallow root systems, which hinder its ability to reach deeper soil layers and extract heavy metals to a treatable level (Słomka et al., 2012). In addition, Wood et al. (2016) observed that non-HMH species often extracted more heavy metals than HMH when measuring the net number of metals extracted per plant, with the number of extracted heavy metals closely correlated to plant biomass. Although HMH remains valuable in practical phytoremediation, non-HMH species with high biomass may represent a more suitable option for developing efficient phytoextractors in the future.

Leguminosae, with 172 genera, 1,485 species, and 153 varieties in China (Hei et al., 2019), are widely distributed throughout the country and hold significance in soil improvement and ecological restoration (Dary et al., 2010; Cai et al., 2015). Legumes have exhibited excellent tolerance and effectiveness in heavy metal remediation, with some species exhibiting remediation capabilities comparable to HMH due to their robust root biomass (Shi et al., 2012; Guo and Chi, 2017; Zeng, 2017). The advantages of legumes in this regard include: (i) their strong root biomass can produce abundant secretions that confer resistance to heavy metal pollution stress (Pereira et al., 2006), with the dissolution of insoluble heavy metals in the soil by the organic acids released from the roots, which can enhance plant uptake of soil heavy metals. (ii) Legume roots contain abundant rhizobia, effectively improving soil quality (Maynaud et al., 2013). These traits enable legumes to enhance, maintain, and develop stable soil systems. Therefore, employing legumes in phytoremediation holds significant potential for soil heavy metal remediation, contributing to the future refinement of plant species screening and phytoextraction in ecological restoration.

In order to improve phytoextraction efficiency, the inoculation of characteristic microorganisms into plant roots is a common strategy (Abhilash et al., 2012; Sessitsch et al., 2013). The identification and development of new, effective PGPR strains would be very efficient, providing several beneficial activities such as improved nutrient uptake, improved stress tolerance, enhanced plant growth, and resistance to fungal or bacterial pathogens (Xiao C. Q. et al., 2021). The phytoremediation coupled with Pb-resistant phosphate-solubilizing bacteria effectively improved the efficiency of Pb bioremediation. For example, The inoculation of soil with strain LA greatly promoted the growth of ryegrass and sonchus, increased the concentration of bioavailable P and Pb in plants, and decreased the bioavailability of Pb in the soil (Guo et al., 2021). In the “biotrophic bacteria-plant” mechanism, on the one hand, “bacteria” generally refers to inter-root biotrophic bacteria that can promote plant growth or improve soil quality, and the microorganisms promote plant growth through the production of iron carriers, phytohormones, organic acids and functional enzymes to enhance the remediation of heavy metals in the soil; On the other hand, some microorganisms themselves have the ability to dissolve and activate heavy metals, which reduces the content of heavy metals in the soil, increases the base of heavy metals that can be absorbed by the soil, and improves the possibility of plant uptake of heavy metals in the soil, so that the total amount of heavy metals in the soil is reduced to a harmless level (Ni et al., 2019). Several studies have demonstrated the microorganisms can convert Cr (VI) in soil into Cr (III) or directly adsorb it in their bodies by means of bioreduction and biosorption, while plants uptake and accumulate Cr in tissues, thus reducing the total Cr content in the soil (Wu et al., 2014). Li (2021) inoculated *Variovorax paradoxus* DE5 into *Celosia argentea* plants and found that DE5 was able to significantly promote the growth of *Celosia argentea* plants and enhance the uptake of soil cadmium by *Celosia argentea*. The results of the pot test showed that the application of *Lactobacillus casei* at 105 cfu·mL^−1^ reduced the pH of the soil, increased the soil enzyme activity, promoted the growth and development of cabbage mustard, and facilitated the remediation efficiency of cabbage mustard on Cd and Zn composite contaminated soil (Liu, 2022). It has been shown that the application of organic acid-secreting endophytic bacteria effectively increased the conversion of insoluble Pb into the effective state of Pb, increased the content of soil heavy metal Pb in the effective state, and significantly improved the enrichment efficiency of oilseed rape for soil heavy metal Pb (Ma Y. et al., 2022). Typically, the candidate microorganisms inoculated into plants originate from internal plant tissues or root secretions and function as “probiotics,” directly or indirectly influencing plant growth (Li et al., 2017). However, such microorganisms often exhibit weak resistance against external pollution stress (Zhang et al., 2011). Conversely, microorganisms exposed to and surviving in heavy metal-contaminated soil over extended periods may possess higher tolerance and resistance (Ibiang et al., 2020). Therefore, understanding the source of microorganisms, their impact on heavy metal morphology, and their probiotic effects on plants prove beneficial for soil heavy metal remediation efforts.

Our study addressed these limitations through a comprehensive approach. We first screened lead (Pb)- and cadmium (Cd)-tolerant microorganisms (resistant bacteria) for inoculum in experiments, using long-term heavy metal-contaminated soil as substrate. In addition, the heavy metal environment was simulated through indoor resistance growth experiments. Concurrently, outdoor pot experiments were conducted to investigate the impact of legumes (*Sophora xanthantha* and *Robinia pseudoacacia* L.) and resistant bacteria-legume combinations on the remediation of heavy metal-contaminated soil. The objective of this study was (a) to determine whether tolerant bacterial inoculum would enhance the effectiveness of legume-based remediation, (b) to examine whether tolerant bacterial inoculum would lead to plant improvement in soil physicochemical properties and reduction in heavy metal content, and (c) to investigate key species of contaminated soil bacterial communities using high-throughput sequencing techniques. These studies have provided essential data and technical support for the utilization of legumes in the remediation of Cd and Pb in soils and the rehabilitation of mining sites.

## 2. Materials and methods

### 2.1. Study area overview and experimental design

**Figure 1:**
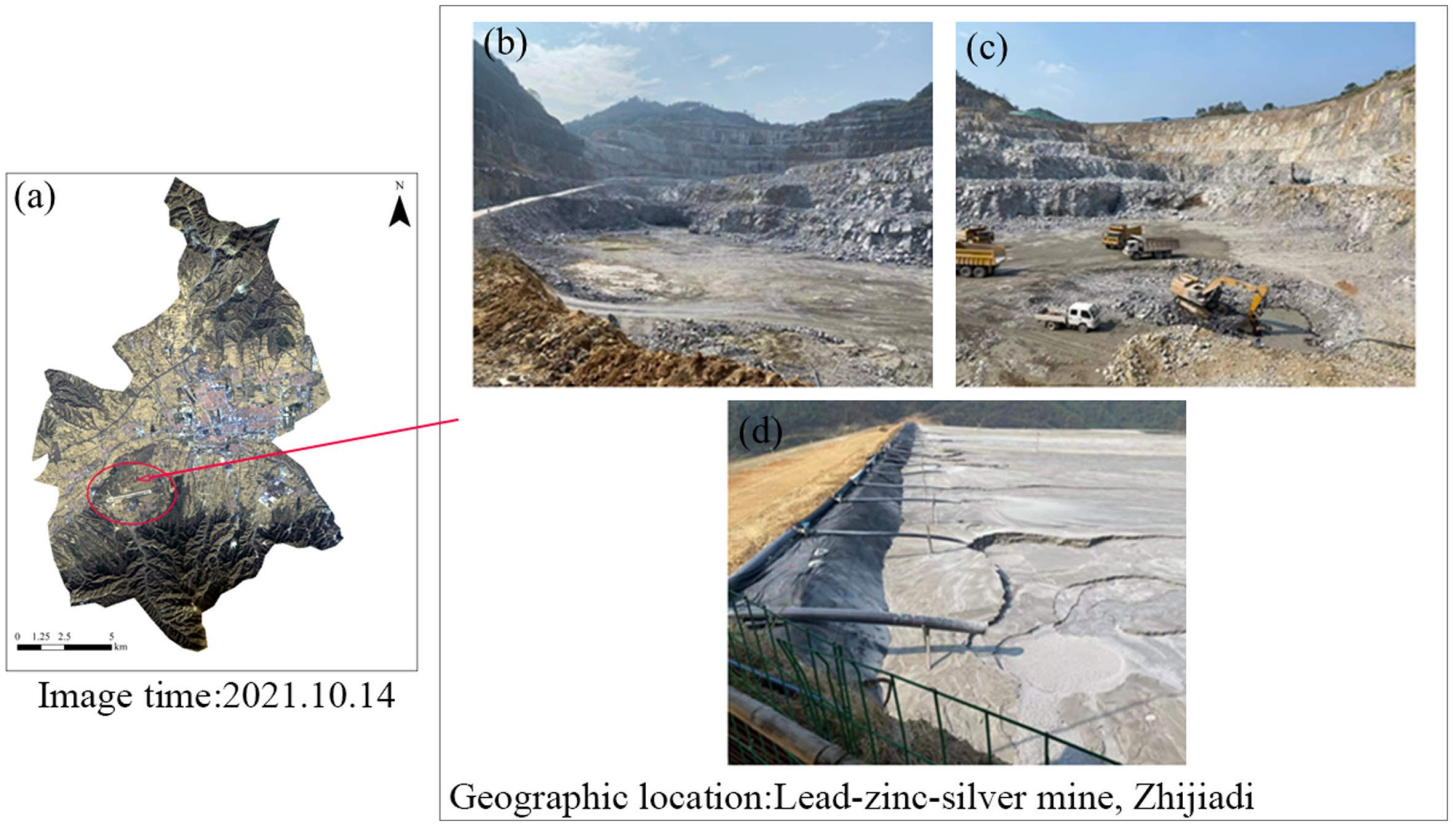
Collection of three different functional types of soils **(a)**, including mine center soils **(b)**, mine wasteland soils **(c)**, and tailings soils **(d)**.

The lead-zinc-silver mine is situated in Zhijiadi, Gaojiazhuang Township, south of Lingqiu County, Shanxi Province (39.21°N, 114.12°E). The region experiences a temperate semi-arid continental climate, with an average annual temperature of 7°C and an average annual rainfall of 421 mm. The mine, commissioned in 2003, is expected to have a lifespan of 20 years or more. Long-term mining activities have led to long-term heavy metal pollution in the soil. In order to address this issue, soil samples were collected from three different functional types: mine center, mine wasteland, and tailings. These soils were used for screening heavy metal Pb and Cd tolerant microorganisms (Figure 1). The experiment involved soils contaminated with two heavy metal complexes, Pb and Cd. Sterilized soil was uniformly sprayed with a solution of 400 mg/L Pb^2+^ (Pb (NO_3_)_2_) and 60 mg/L Cd^2+^ (CdCl_2_). After thorough mixing, the soil was left for 15 days for potting experiments.

#### 2.1.1. Resilient growth experiment

Bacteria and fungi were isolated from soil samples using 10-fold serial dilutions plated onto heavy metal-free nutrient agar and potato dextrose agar solid media, respectively. The plates were then incubated for 3 days at 28°C. Once colonies covered two-thirds of the plates, colonies indicating different morphological colors were selected and purified. Subsequently, solid medium plates containing a Pb^2+^ concentration of 100 mg/L and a Cd^2+^ concentration of 40 mg/L were prepared. The purified strains were plated on these plates using the “trilinear” method. The screening of strains that grew well on plates containing heavy metals for resistance growth experiments was conducted to identify strains capable of thriving in environments contaminated with lead and cadmium while reducing unnecessary testing efforts.

The viability of the screened strains was quantitatively assessed through resistance growth experiments. Luria broth (LB) cultures with varying concentrations of Pb and Cd (suitable for bacterial and fungal colonization) were prepared. The Pb concentrations in the LB cultures were set at 400, 600, 800, 1,000, 1,500, and 2,000 mg/L, achieved by replacing pure water with different concentrations of Pb (NO_3_)_2_ solution. Similarly, the Cd^2+^ concentrations in LB cultures were maintained at 50, 100, 150, 200, 250, and 300 mg/L. Well-grown colonies (WZ13, WZ14, WG20, and S22) with a diameter of 1 mm were added to 500 mL conical flasks containing 400 mL LB culture medium with varying Pb and Cd concentrations. Control flasks without colonies were also included. The flasks were then incubated on a shaker (speed: 180 rpm, temperature: 25°C ± 0.3) for 2 days. During this period, absorbance values were measured at 12, 24, 36, and 48 h for each treatment, providing insights into the strain’s ability to survive in an environment contaminated with lead and cadmium (Fang et al., 2012).

#### 2.1.2. Pot experiment

Based on the results of resistance growth and soil cultivation experiments, a promising resistant strain was selected for investigating the remediation effects of soil Pb and Cd through potting experiments. The seeds of the test plants were provided by the State-owned Qiaotou Forest Farm, Wengniute Banner, Chifeng City, Inner Mongolia. Seeds were soaked in pure water for 12 h (Zheng et al., 2021). After filtering the water and allowing the seeds to dry on the surface, the obtained strain was disinfected by immersing them in a 5% sodium hypochlorite solution for 10 min (Zhuang et al., 2021). After washing the seeds with pure water until they were odorless, 5 to 7 seeds were placed in seedling cups for 1 week. Seedlings of similar height and growth were carefully selected for transplantation into plastic pots (15 cm in diameter, 20 cm in height, containing 2.0 kg of soil mixture; Bao, 2000). Once the seedling roots stabilized, the prepared WZ14 inoculum was inoculated into the plant roots, creating a combination of tolerant bacteria-plant treatment. Seedlings inoculated with sterile culture served as the control treatment. Each treatment was replicated three times and consistently watered and randomized to maintain a standardized regimen. After 3 months, plant and soil samples were collected for analysis.

### 2.2. Sample collection and analysis

After a 3-month growth period, all plants and soil were harvested. Plant samples were collected by separating roots, stems, and leaves, which were then washed, dried, and crushed. Soil samples were obtained by shaking the inter-root soil attached to plant roots. The selected soil samples were divided into two parts and stored. One portion of the soil was stored in a 4°C ice box and brought back to the laboratory for further analysis. The second portion of the soil samples was air-dried to determine the physical and chemical properties of the soil.

The pH of the soil solution and the soil’s physicochemical properties were analyzed following Bao (2000) and Lou et al. (2019) standard method. Potentiometric measurements determined the pH, volumetric potassium dichromate analysis determined soil organic matter, and the elemental analyzer determined total soil nitrogen. The molybdenum-antimony anti-colorimetric method was employed for the determination of total soil phosphorus, while total soil potassium was measured using the NaOH alkali fusion-flame photometric method. Soil alkaline nitrogen was determined via the alkaline diffusion method, and effective phosphorus was analyzed using the molybdenum-antimony anti-colorimetric UV spectrophotometric method. Fast-acting potassium was determined by ammonium acetate extraction followed by flame analysis. The effective phosphorus content was quantified using molybdenum antimony anti-colorimetric UV spectrophotometry. The total amount of heavy metal elements in soil samples was analyzed through soil digestion using the electric hot plate digestion method. Additionally, the effective state content of soil heavy metals was extracted using the hydrochloric acid leaching method (HJ804-2016). Both analyses were conducted using an Optima 5300 DV inductively coupled plasma emission spectrometer test (ICP-AES, PerkinElmer, United States).

1. Measurement of total heavy metals in soil: Soil samples were air-dried and sifted through a 100-mesh sieve. Then, 0.1 g of the soil sample was put in a 25 mL PTFE beaker, lightly moistened with Milli-Qultrapure water and heated at a low temperature of 180°C with 5 mL of concentrated hydrochloric acid for 20 min (evaporated to about 3 mL) to eliminate sulfur compounds present in the sample. Subsequently, 3 mL of concentrated nitric acid, 3 mL of hydrofluoric acid, and 1 mL of perchloric acid were added. The beaker was then heated on a heating plate with a lid to a temperature of 280°C for approximately 1.5 h until a clear solid was produced. Afterward, 19 mL of ultrapure water and 1 mL each of concentrated hydrochloric acid and concentrated nitric acid (concentrated hydrochloric acid: concentrated nitric acid = 3:1) were added to the beaker. Then, the mixture was transferred to a 25 mL volumetric flask, and the volume was determined. Ultimate, taking 5 mL of the solution that has passed through a 0.45 μm aqueous filter membrane and place it in a 10 mL centrifuge tube. For testing.
2. Determination of the effective state content of soil heavy metals: The soil samples were air-dried and passed through a 100-mesh sieve, and 5 g of soil samples were placed in a 15 mL centrifuge tube. Then 0.1 mol-L^−1^ of dilute hydrochloric acid was added to the centrifuge tube, the lid was tightly closed, and the tube was placed on a shaker (speed: 200 rpm, temperature: 20°C ± 2°C) for 4 h to allow the reaction to complete, and the tube was removed from the shaker. Ultimate, after being left standing for 24 h, take 5 mL of the solution that has passed through a 0.45 μm aqueous filter membrane and transfer it to a 10 mL centrifuge tube for testing.

The method for determining heavy metals in different parts of the plant was identical to the one described above for total soil heavy metals.

### 2.3. Soil DNA extraction, PCR amplification, and high-throughput gene sequencing

The PowerSoil DNA Isolation Kit (MO BIO, CA, United States) was utilized to extract DNA from all soil samples. In order to minimize the impact of soil heterogeneity on test results and avoid biases from single DNA extraction or low DNA content in samples, each soil sample underwent multiple DNA extracts for subsequent analysis. The purity and concentration of the DNA were assessed using a NanoDrop-2000 spectrophotometer (Thermo-Scientific, DE, United States) and agarose gel electrophoresis (Li et al., 2020). For soil DNA amplification, specific primers 338F (5′-ACTCCTACGGGAGGCAGCAG-3′) and 806R (5′-GGACTACHVGGGTWTCTAAT-3′) targeting the V3–V4 region of bacterial 16S rRNA were employed (Ma et al., 2020). The PCR amplification steps were as follows: the predenaturation at 95°C for 3 min, followed by 35 cycles at 95°C for 30s, 55°C for 30 s, and 72°C for 45 s, were performed with a final extension at 72°C for 5 min. The total volume of the PCR reaction was 25 μL. After PCR amplification, the constructed libraries were analyzed through Qubit and qPCR before being sequenced on an Illumina Miseq system (Guangzhou Kidio Technology Services). The raw data obtained from sequencing was processed by trimming, filtering, and splicing to obtain valid data for subsequent analysis. The optimized sequences, showing >97% similarity, were clustered into operational taxonomic units (OTUs). The taxonomic identities of the bacteria were determined using RDP software and Silva schemes (Wang et al., 2007; Quast et al., 2012).

For further experiments, the nucleotide sequence of WZ14 and the ITS sequence of the potting soil have been uploaded to the NCBI database under the accession numbers OR492361 and PRJNA1012100, respectively.

### 2.4. Data analysis

Data were compiled using Microsoft Excel 2019 software, and statistical analyses were conducted using one-way analysis of variance (ANOVA) with SPSS 20.0 (IBM, United States) for total soil heavy metals, effective soil heavy metal status, and soil physicochemical properties. Statistical significance was accepted at *p* < 0.05. To address soil microbial diversity and richness variations, Simpson and Shannon diversity indices, as well as Chao1 and ACE richness indices, were calculated using Mothur and analyzed for alpha diversity. The data were plotted using the Origin 2019 software, and the significance of differences was determined using the DUNCAN method (α = 0.05). Additionally, heat maps were generated to illustrate the correlation between soil physicochemical factors and soil heavy metals. All bioinformatics analyses were performed using the Omicsmart online analysis platform developed by Guangzhou Kidio Technology Services.

## 3. Results and analysis

### 3.1. Resistance growth and soil culture trials based on microbial screening

In this study, we isolated a total of 70 strains from the mine center, mine wasteland, and tailings. A Pb^2+^ concentration of 100 mg·kg^−1^ and a Cd^2+^ concentration of 40 mg·kg^−1^ were used as screening thresholds for microbial adaptation to heavy metal environments. Among the strains tested, 26 exhibited the ability to tolerate heavy metals. Following a qualitative evaluation, we selected four strains with excellent lead and cadmium tolerance, which were named WZ14, WZ13, S22, and WG20. These selected strains were further subjected to resistance growth experiments.

**Figure 2:**
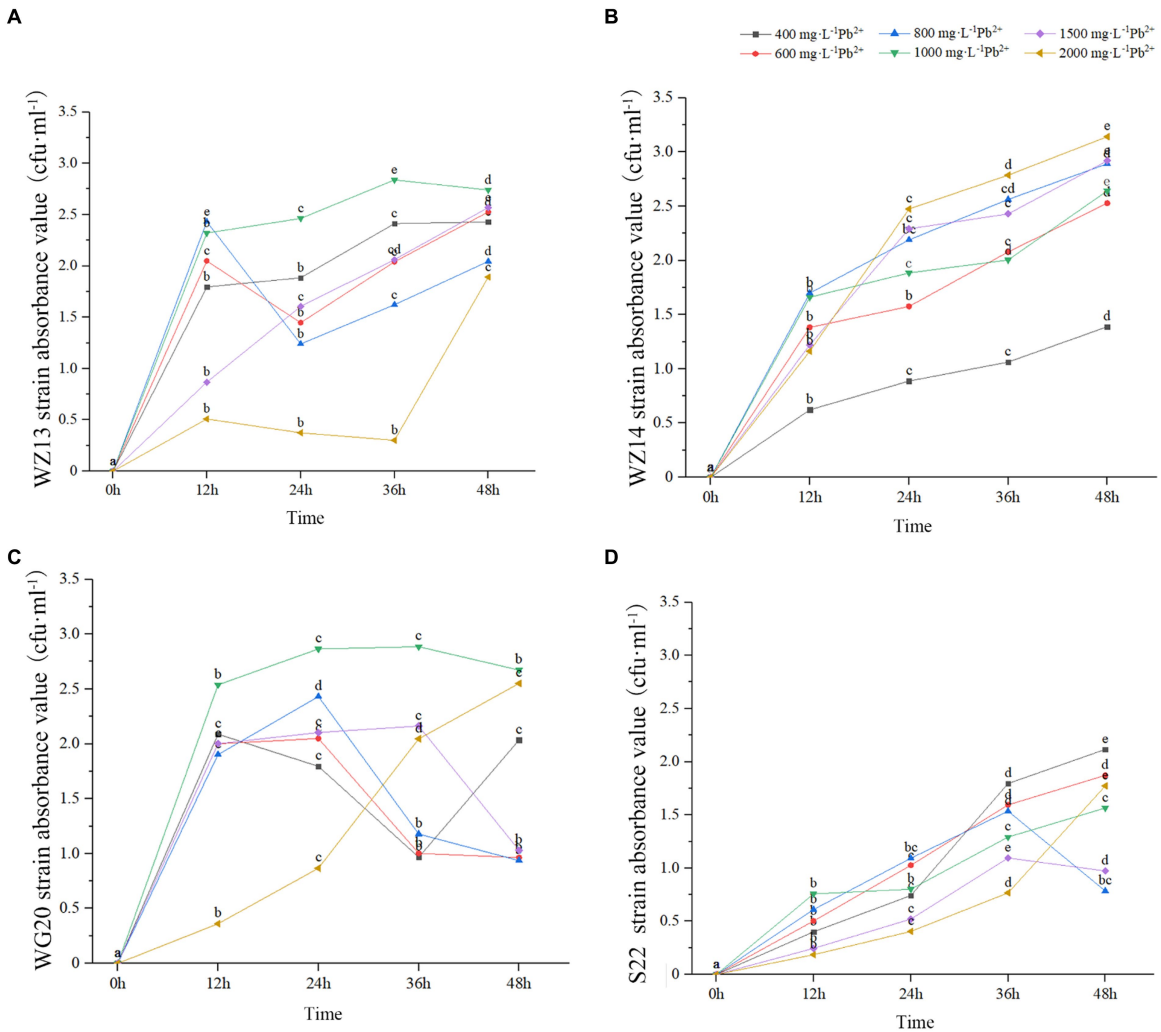
Growth of four strains in medium with different concentrations of Pb^2+^. Different lowercase letters indicate significant differences between concentrations (*p* < 0.05). WZ13 strain **(A)**; WZ14 strain **(B)**; WG20 strain **(C)**; S22 strain **(D)**.

**Figure 3:**
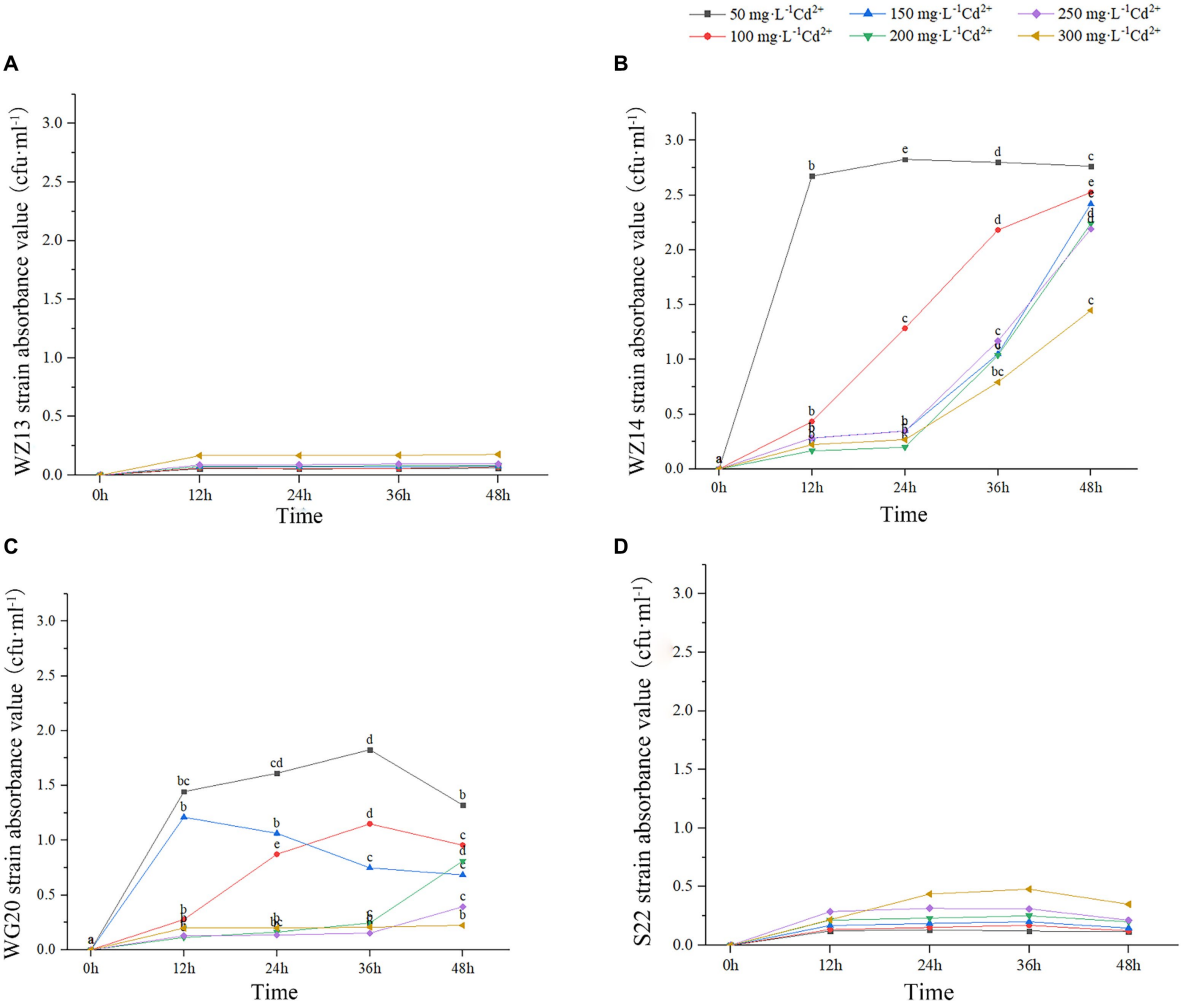
Growth of four strains in medium with different concentrations of Cd^2+^. Different lowercase letters indicate significant differences between concentrations (*p* < 0.05). WZ13 strain **(A)**; WZ14 strain **(B)**; WG20 strain **(C)**; S22 strain **(D)**.

The four strains exhibited varying growth responses to cultures with different Pb^2+^ and Cd^2+^ concentrations (Figures 2, 3). Each strain displayed distinct sensitivity levels to the same heavy metal. Notably, WZ14 exhibited the most favorable adaptation to different Pb^2+^ concentrations without showing a peak in the growth curve for each concentration. It differed from other strains in cultures with different Pb^2+^ concentrations. WZ13 and S22 demonstrated weak acclimation in Cd^2+^ cultures, with both strains exhibiting growth of no more than 0.5 cfu·mL^−1^ in various Cd^2+^ concentrations, significantly lower than that of WZ14 and WG20. Comparatively, WZ14 displayed a higher growth curve than WG20 at the same Cd^2+^ concentration. In addition, its curve gradually shifted downward as Cd^2+^ concentration increased, suggesting the suitability of WZ14 for survival in a low-concentration Cd-contaminated environment. The results from the resistance growth experiment indicated that WZ14 exhibited robust growth and excellent resistance in both Cd- and Pb-contaminated environments, outperforming other strains.

WZ14 exhibited the highest resistance and most effective reduction in heavy metal content than other three strains, leading to its selection for the subsequent tests.

### 3.2. Influence of applied microbial inoculants on nutrient uptake and accumulation of Pb and Cd by plants

*Robinia pseudoacacia* monoculture, WZ14-*R. pseudoacacia*, *S. xanthantha* monoculture, and WZ14-*S. xanthantha* significantly increased soil pH compared to the soil background (BJZ; Table 1). However, when WZ14 was applied to *R. pseudoacacia* and *S. xanthantha*, it slightly reduced the soil pH compared to plant monocultures. All four treatments significantly increased the organic matter content (OC) of the soil compared to BJZ. WZ14-*R. pseudoacacia* and WZ14-*S*. *xanthantha* showed a significant difference, with an increase of 14.36% and 18.29%, respectively, compared to no strain application. For soil alkaline nitrogen content, all treatments significantly reduced it compared to BJZ. *Robinia pseudoacacia* and *S. xanthantha* significantly increased soil alkaline nitrogen content by 79.66% and 61.04%, respectively, after applying the WZ14 strain compared to no strain application. All four treatments significantly reduced the soil’s effective phosphorus content compared to BJZ, with *R. pseudoacacia* having the lowest soil effective phosphorus content significantly after the application of the WZ14 strain. The strain-legume combinations significantly reduced soil fast-acting potassium content compared to plant monocultures with WZ14-*R. pseudoacacia* showing the most significant reduction. Regarding allotropic nutrients, compared to plant monocultures, both *R. pseudoacacia* and *S. xanthantha* showed increased soil allotropic nitrogen, phosphorus, and potassium content after applying the WZ14 strain, indicating a higher potential for allotropic nutrients to transform into effective nutrients that can be easily absorbed by plants. However, the application of WZ14 also caused irregular changes in soil effective nutrient content, influenced by both the decomposition of insoluble nutrients and the absorption capacity of surface plants.

**Table 1:** Effects of different treatments on soil physicochemical properties.

Soil physicochemical	BJZ	*Robinia pseudoacaca*	WZ14-*R. pseudoacacia*	*Sophora xanthantha*	WZ14-*S. xanthantha*
pH	6.90 ± 0.08a	6.98 ± 0.02b	6.95 ± 0.02b	6.99 ± 0.02b	6.96 ± 0.02b
OC (mg/kg)	11.54 ± 0.50a	12.88 ± 0.43ab	14.73 ± 0.27c	13.83 ± 0.72bc	16.36 ± 0.86d
TN (g/kg)	2.31 ± 0.03e	2.09 ± 0.02d	1.74 ± 0.01a	1.96 ± 0.01c	1.87 ± 0.02b
TP (g/kg)	0.87 ± 0.03b	0.83 ± 0.03ab	0.79 ± 0.02a	0.80 ± 0.01a	0.78 ± 0.03a
TK (g/kg)	23.14 ± 0.31a	25.65 ± 0.27d	24.80 ± 0.30c	26.30 ± 0.25d	23.85 ± 0.31b
AN (g/kg)	203.46 ± 1.69d	74.05 ± 2.33a	133.04 ± 3.54c	84.98 ± 2.30b	136.85 ± 1.56c
AP (mg/kg)	114.50 ± 0.43d	96.38 ± 0.05c	87.48 ± 0.14b	86.94 ± 5.62b	71.37 ± 0.07a
AK (mg/kg)	247.90 ± 2.84e	153.90 ± 3.49c	117.90 ± 4.03a	163.84 ± 3.57d	132.71 ± 3.50b

BJZ represents soil physicochemical property values before the pot experiment. Different lowercase letters indicate significant differences between treatments (*P* < 0.05).

**Figure 4:**
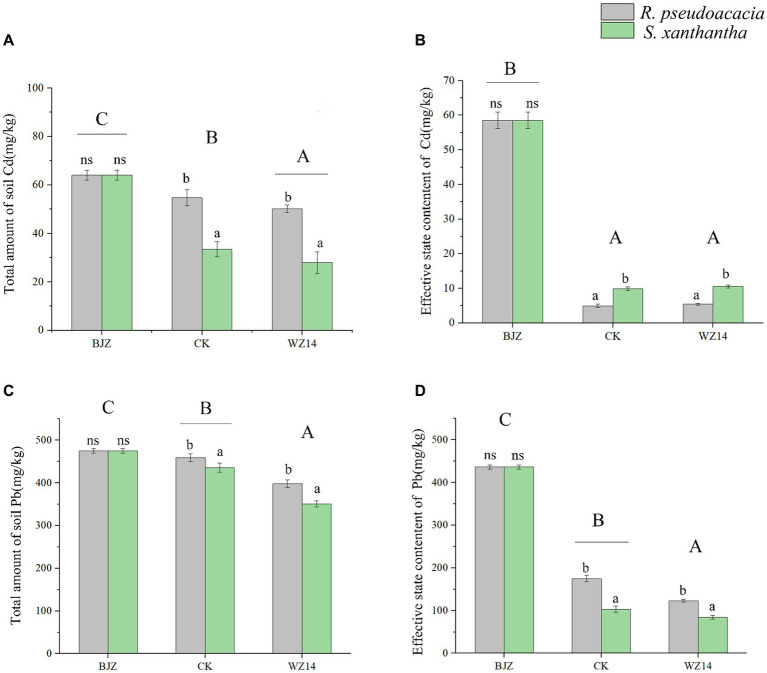
Effect of different treatments on total soil heavy metals Cd **(A)** and Pb **(C)** and effective state content of Cd **(B)** and Pb **(D)**. BJZ, background value of soil before pot experiment; CK, no application of microbial agent; WZ14, the application of WZ14 microbial inoculum. Different lowercase letters indicate significant differences between different plants in the same treatment (*p* < 0.05). Different uppercase letters indicate significant differences between treatments (*p* < 0.05). ns: indicates no significant differences between different plants in the same treatment (*p* < 0.05).

After phytoremediation and combined remediation, the soil Pb and Cd contents were significantly lower than the soil heavy metal contents before potting (Figure 4; *p* < 0.05). However, the effectiveness of the two legumes in remediating Pb- and Cd-contaminated soil differed after the application of the WZ14 strain. Compared to the single plant treatments, the combined WZ14-*R. pseudoacacia* and WZ14-*S*. *xanthantha* combinations reduced total soil Pb by 13.33% and 19.45%, respectively, and reduced the effective state of soil Pb by 29.63% and 18.11%, respectively. In addition, the WZ14-*R. pseudoacacia* combination treatment significantly reduced the soil Cd total and Cd active state content by 20.37% and 90.72%, respectively, compared to the soil heavy metal content before potting (Figure 4; *p* < 0.05). The WZ14-*S*. *xanthantha* combination treatment significantly reduced the soil Cd total and Cd active state content by 55.72% and 82.71%, respectively. Regarding the reduction of soil Cd effective state, the plant monoculture treatments of *R. pseudoacacia* and *S. xanthantha* showed a reduction of 91.51% and 83.14% in soil Cd effective state, respectively, compared to the soil background values before potting. The WZ14-legume combinations were more effective than single-crop treatments in reducing soil Cd levels but were weaker than single-crop treatments in effectively remediating soil Cd status.

**Figure 5:**
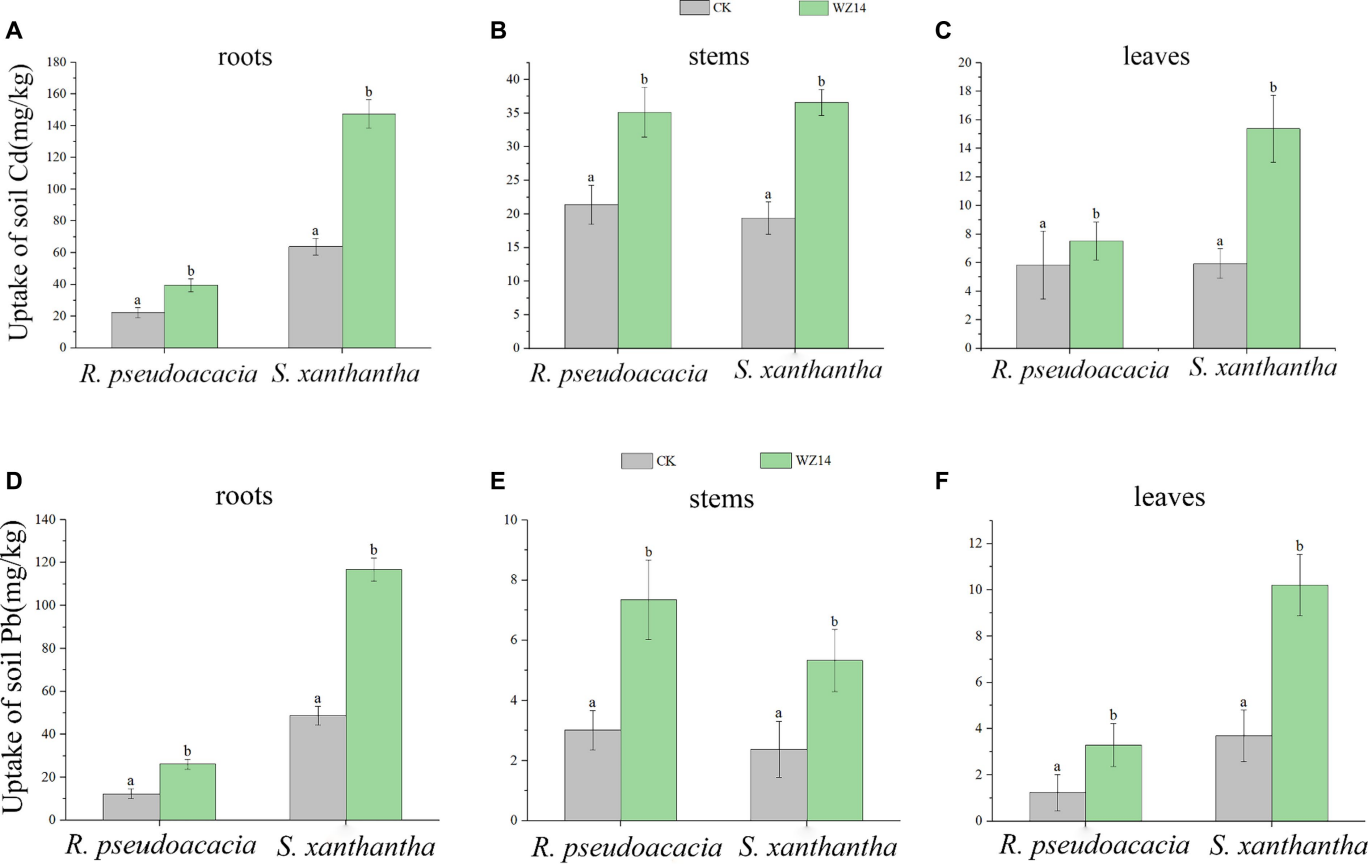
Uptake of soil heavy metals Cd **(A–C)** and Pb **(D–F)** by roots, stems, and leaves of *Robinia pseudoacacia L.* and *Sophora japonica*. Different lowercase letters indicate significant differences between treatments (*p* < 0.05).

The Pb and Cd contents in the roots of *S. xanthantha* were significantly higher than in the CK (plant monoculture treatment) and *R. pseudoacacia* treatments under bacterial addition. In contrast, the Pb content in the stem of the WZ14-*R. pseudoacacia* treatment was significantly higher than in other treatments (Figure 5, *p* < 0.05). For *R. pseudoacacia* plants, Pb content in roots, stems, and leaves was generally low, ranging from 12.37 to 26.07, 3.01 to 7.35, and 1.24 to 3.29 mg·kg^−1^, respectively. However, when combined with WZ14, the Pb content significantly increased in WZ14-*R. pseudoacacia* roots by 110.8% and in stems and leaves by 144.2% and 165.3%, respectively, compared to *R. pseudoacacia* monoculture (Figure 5, *p* < 0.05). In the case of Cd content, there was no significant difference in the stems of the two plants under the strain application treatment, and both Pb and Cd content in *R. pseudoacacia* roots were significantly lower than in *S. xanthantha* under monoculture (*p* < 0.05). Cd content was higher in *S. xanthantha* plants, and under the strain application treatment, Cd content in roots, stems, and leaves increased significantly by 130.79%, 88.64%, and 159.13%, respectively, compared to CK (*p* < 0.05). Pb content in *S. xanthantha* stems and leaves also increased significantly by 124.9% and 157.8%, respectively, compared to CK (*p* < 0.05). In comparing the uptake capacity of Pb and Cd, it was observed that the Pb and Cd contents in roots and leaves were significantly higher in the combined WZ14-*S. xanthantha* treatment than in the combined WZ14-*R. pseudoacacia* treatment. However, in stems, the Pb content was lower in the combined WZ14-*S. xanthantha* treatment than in WZ14-*R. pseudoacacia*, and there was no significant difference in the Cd content between the two (*p* > 0.05). This pattern was consistent with the single plant treatment, indicating that *S. xanthantha* (except for the stem) had a better capacity for soil Pb and Cd uptake than *R. pseudoacacia* (except for the stem).

### 3.3. Impact of the added bacterial agent on the inter-rhizosphere soil bacterial community

Overall, the addition of the WZ14 strain had varying effects on soil bacterial alpha diversity in *R. pseudoacacia* and *S. xanthantha* (Table 2). In *R. pseudoacacia*, the presence of the WZ14 strain increased the abundance and diversity of soil microbial communities compared to the plant treatment without the WZ14 strain. However, in *S. xanthantha* soil, the addition of the WZ14 strain significantly reduced the abundance of OTUs and the diversity of bacterial communities, with the Chao1 index and Shannon index decreasing by 9.86% and 6.45%, respectively, compared to *S. xanthantha* monoculture.

**Table 2:** Difference of alpha diversity index of soil bacteria under different treatments.

Different treatments	Abundance index		Diversity index		Sequencing depth Index
	ACE index	Chao1 index	Simpson index	Shannon index	Coverage
*Robinia pseudoacacia*	3096.85 ± 12c	2919.08 ± 22c	0.991 ± 0.001a	8.725 ± 0.013b	0.991
WZ14-*R. pseudoacacia*	3130.05 ± 8d	2943.37 ± 11d	0.990 ± 0.001a	8.766 ± 0.009d	0.988
*Sophora xanthantha*	2881.66 ± 10b	2826.08 ± 9b	0.988 ± 0.001a	8.745 ± 0.021c	0.994
WZ14*-S. xanthantha*	2601.58 ± 9a	2547.43 ± 13a	0.986 ± 0.02a	8.181 ± 0.019a	0.986

Different lowercase letters indicate significant differences between treatments (*P* < 0.05).

**Figure 6:**
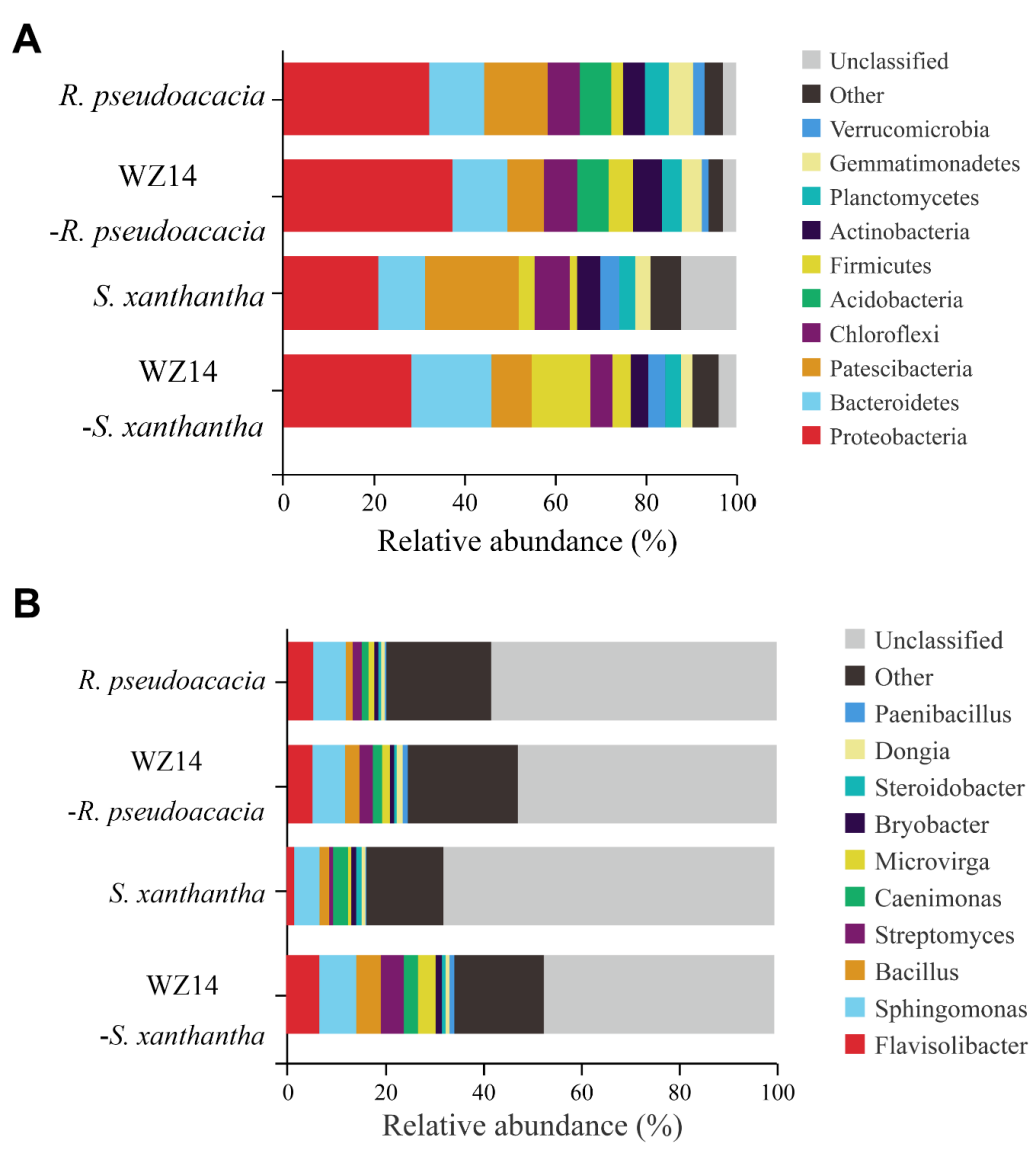
Effects of different treatments on the relative abundance of major phyla **(A)** and genera **(B)** of soil bacterial communities.

At the phylum level (Figure 6A), the dominant phyla of the bacterial community in the soil samples were *Proteobacteria*, *Bacteroidetes*, *Patescibacteria*, *Chloroflexi*, and *Acidobacteria*, each representing the relative abundance of more than 5% of the total soil bacterial community in the upper soil, accounting for 64.48% to 72.61%. After inoculation with WZ14, the relative abundances of *Proteobacteria* and *Firmicutes* in the soil of *R. pseudoacacia* and *S. xanthantha* significantly increased after inoculation with WZ14 compared to the treatment without microbial inoculants. Specifically, the relative abundance of *Proteobacteria* in the soil of *R. pseudoacacia* increased from 32.76% to 37.42% and in *S. xanthantha* from 20.92% to 29.78%, while *Firmicutes* increased from 2.60% to 5.35% in *R. pseudoacacia* and from 3.52% to 6.88% in *S. xanthantha*. Additionally, WZ14 inoculation significantly increased the relative abundance of *Bacteroidetes* from 10.28% to 17.91% (*p* < 0.05) and reduced the relative abundance of *Patescibacteria* from 13.51% to 8.01% in *R. pseudoacacia* and from 20.49% to 10.18% in *S. xanthantha* soils, respectively. These findings suggested that WZ14 inoculation may play a vital role in improving plant quality and remediating heavy metal contamination by increasing the abundance of dominant bacterial communities, particularly the relative abundance of *Proteobacteria*, *Bacteroidetes*, and *Firmicutes*.

At the genus level (Figure 6B), the dominant genera in the soil samples were *Flavisolibacter*, *Sphingomonas*, *Bacillus*, *Streptomyces*, and *Paenibacillus*. The addition of WZ14 significantly increased the relative abundance of the dominant bacterial genera (total relative abundance of the five most abundant genera). Specifically, the relative abundances of *Flavisolibacter*, *Sphingomonas*, and *Bacillus* increased from 1.79%, 5.19% and 1.98% to 7.02%, 7.57%, and 5.03%, respectively, after the addition of WZ14. However, the relative abundance of *Paenibacillus* in the soil was significantly reduced. Similar trends were observed in the abundance of *Bacillus* and *Streptomyces* in *R. pseudoacacia* soils, with relative abundances increasing from 1.43% and 1.82% to 2.96% and 2.71%, respectively, after WZ14 application. However, the application of WZ14 did not significantly change the abundance of *Sphingomonas* and *Flavisolibacter* in *R. pseudoacacia* soils.

### 3.4. Correlation between inter-root bacterial communities and soil environmental factors

Spearman’s correlation analysis was used to clarify the relationship between soil bacterial communities and soil physicochemical factors in *R. pseudoacacia* and *S. xanthantha* after WZ14 inoculant application at the phylum and genus levels.

**Figure 7:**
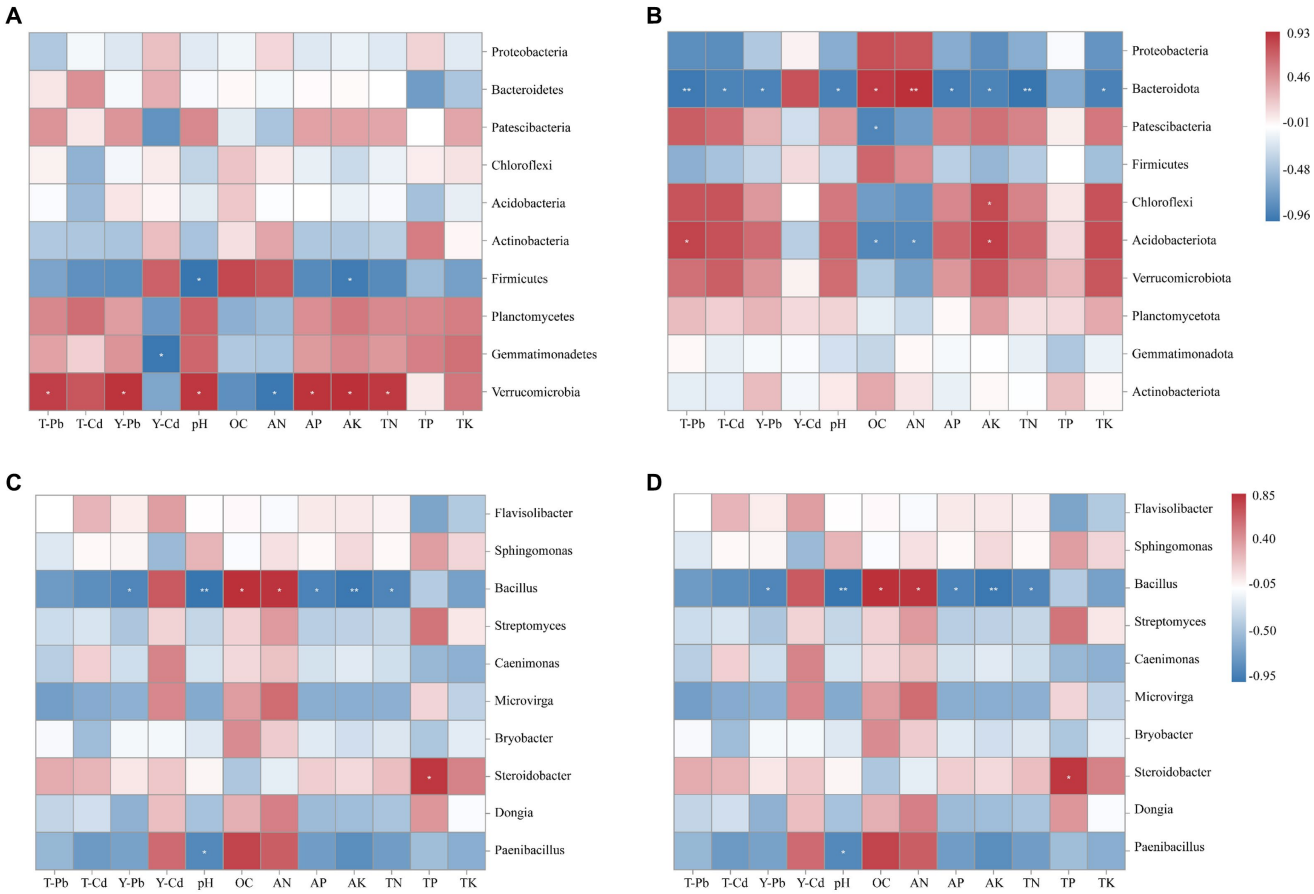
Spearman analysis between soil physicochemical properties in *Robinia pseudoacacia* **(A,C)** and *Sophora xanthantha* **(B,D)** and soil bacterial community at phylum and genus levels. Color represents R value; * indicates *p* < 0.05; ** indicates 0.05 ≤ *p* ≤ 0.01; *** indicates *p* < 0.01.

According to Spearman’s correlation heat map (Figure 7), *Verrucomicrobia* exhibited significant positive correlations with soil pH, AK, AP, and TN while showing a significant negative correlation with soil solution AN content. On the other hand, *Firmicutes* showed a significant negative correlation with soil pH and AK. Additionally, *Bacteroidota* showed significant positive correlations with OC and AN and negative correlations with soil pH, AP, AK, TN, and TK in *S. xanthantha* soils. *Acidobacteriota*, in the same soils, demonstrated significant negative correlations with OC and AN while positively correlating with AK. At the genus level, environmental factors appeared to mainly affect *Bacillus*, *Steroidobacter*, and *Paenibacillus* in *R. pseudoacacia*. For example, *Bacillus* showed significantly negative correlations with soil pH, AP, AK, and TN, but positive correlations with OC and AN. TP exhibited a significant positive correlation with *Steroidobacter*, and pH showed a negative correlation with *Paenibacillus*. *Flavisolibacter* in *S. xanthantha* soils showed highly significant negative correlations with AK and TK while exhibiting positive correlations with OC and AN. *Microvirga* showed significant negative correlations with AK and TK and positive correlations with OC and AN. *Steroidobacter* indicated significant negative correlations with pH, AK, TN, and TK and positive correlations with AN. This study identified that soil Pb content was significantly and positively correlated with *Verrucomicrobia*, but negatively correlated with *Bacteroidota*. The effective state of soil Cd was significantly and negatively correlated with *Gemmatimonadetes*, while the total soil Cd was significantly and negatively correlated with *Bacteroidota*.

## 4. Discussion

### 4.1. Effect of soil physicochemical properties under combined remediation

Soil physicochemical properties are fundamental indicators used to characterize soil nutrients, which in turn determine the overall fertility level of the soil. The presence of volatile nutrients, susceptible to environmental influences, directly reflects soil quality (Glick, 2010). In this context, microorganisms act as essential “regulators” of geobiochemical processes, playing a vital role in maintaining soil vitality and ecological functions (Liu et al., 2023).

The total soil nutrient content, including total N and total P, and fast-acting nutrients demonstrated a decrease but without significant difference, consistent with the findings of Hussain et al. (2018). Soil nutrient loss is directly related to heavy metal contamination, with higher contamination levels leading to more significant loss of soil nitrogen, phosphorus, potassium, and fast-acting nutrients. Moreover, in this study, the observed changes in soil phosphorus content after applying mycorrhizal fungi can be attributed to the enhancement of plant nutrient uptake by these fungi. This, in turn, promoted plant growth and optimized the plant’s ability to remediate heavy metals through phosphorus solubilization (Table 1; Guo et al., 2013; Hu et al., 2019). Soil pH changes can directly affect the effective levels of soil nutrients, microbial activities, and the toxic effects of heavy metal ions. Under Cd and Pb contamination, both monoculture and combined remediation improved the soil organic carbon levels to some extent, with the combined system of inoculated bacteria and legumes showing the best results. However, propagating plants under heavy metal contamination reduced the alkaline-dissolved nitrogen levels of the soil. The inoculation of bacteria in plant monocultures inhibited the declining impact of plants on soil alkaline digestion of nitrogen. It also mitigated the decline in soil alkaline-dissolved nitrogen, consistent with the findings of Cui (2019) and Hussain et al. (2018). This may be attributed to the ability of inoculation treatment to enhance the accumulation of soil carbon and nitrogen nutrient to varying degrees, promote plant root growth, and improve the antagonistic ability of plants.

### 4.2. Effect of soil Pb and Cd uptake under combined remediation

Heavy metals in soil exist in various forms. The exchangeable form of heavy metals in soil is highly mobile and easily absorbed by plants, unlike the organic, carbonate, and ferro-manganese oxidation forms, which are less readily absorbed. Determination of the effective state content of heavy metals is an effective way to determine the extent of heavy metal contamination and to predict the impact of heavy metals on ecosystems (Zhou et al., 2017). Therefore, the primary objective of remediation is to reduce the amount of active heavy metals in the soil, which in turn affects their crop uptake (Wang et al., 2020). Numerous studies have demonstrated that the interaction between plant and microorganisms can enhance plant biomass and improve heavy metal tolerance, facilitating the absorption, fixation, and reduction of heavy metal concentrations in the soil. This process reduces the toxic effects of heavy metals (Khan et al., 2017; Fu et al., 2022; Zheng et al., 2022a). In this study, the combined treatment with WZ14 was more effective than individual plant treatments. The roots of *R. pseudoacacia* and *S. xanthantha* showed significantly higher levels of Pb and Cd than stems and leaves, which were the primary sites of soil Pb and Cd uptake. This difference was due to the limited translocation capacity of non-enriched plants, leading to the accumulation of Pb and Cd in their roots. These findings aligned with previous research (Sun and Mao, 2015).

Microorganisms in the soil play a crucial role in both the formation of soil humus and the mineralization of organic matter, the uptake and accumulation of soil Pb and Cd by *R. pseudoacacia* and *S. xanthantha* were strongly influenced by soil properties and fertility. In this study, the addition of WZ14 bacterial agent increased the OC content of soils occupied by *R. pseudoacacia* and *S. xanthantha*. Additionally, it enhanced the metabolic processes of inter-root microorganisms and their byproducts. These changes influenced the migration and release mechanisms of Pb and Cd, leading to reduced toxicity of heavy metals in the soil.

### 4.3. Correlation analysis of soil environmental factors and soil bacterial communities

Strong correlations exist between plant and soil microbial communities, with plant root secretions and residues influencing the function and structure of soil microbial communities (Bian et al., 2018). In this study, the addition of WZ14 bacterial agent had contrasting effects on soil bacterial communities of *R. pseudoacacia* and *S. xanthantha*. Although it enriched the richness and diversity of soil bacterial communities in *R. pseudoacacia*, it reduced the richness and diversity in *S. xanthantha*. These changes could be due to short-term responses of bacterial communities to environmental and pollution changes, reflecting their microenvironmental conditions (Yao et al., 2019). Although the exogenous soil microorganism WZ14 partially promoted the development of heavy metal-tolerant microorganisms, it also intensified competition among soil microorganisms. Consequently, less adaptable microorganisms struggled to cope with environmental changes and experienced a decline in abundance and diversity (Zhang et al., 2020).

The majority of bacterial communities in this study showed a significant negative correlation with soil physicochemical factors, possibly due to the ability to decompose and utilize soil nutrients for energy, either for themselves or plants (Yao et al., 2022). The results of this study revealed that Proteobacteria had the highest relative abundance in both monoculture and grafted-plant combination models, indicating the prevalence of Proteobacteria in heavy metal-contaminated soils and their tolerance to high levels of Cd and Pb contamination (Gupta et al., 2017). Proteobacteria emerged as the dominant phylum in the heavy metal-contaminated soil of the mining area and positively contributed to improving soil contamination status. However, its relative abundance was negatively correlated with the overall soil quality (Yu et al., 2020; Ma J. D. et al., 2022). In contrast, our study discovered the highest abundance of Proteobacteria in the soil of *R. pseudoacacia* and *S. xanthantha* when the heavy metal contamination level decreased after the application of the microbial inoculant. This discrepancy may arise from the specific focus of our experiment on a combined Pb and Cd contamination pattern, resulting in different responses of bacterial communities to Pb and Cd under heavy metal-contaminated conditions.

## 5. Conclusion

Microorganisms residing in heavy metal environments for extended periods have developed a notable tolerance and resistance to heavy metal ions. In order to address heavy metal contamination in soil, one of the effective approaches can be exploring heavy metal-resistant bacteria for their capacity to absorb and accumulate heavy metals (Zheng et al., 2022b). In this study, we successfully isolated and purified an efficient Cd- and Pb-tolerant strain from the contaminated soil of lead-zinc-silver mine in Zhijiadi, Shanxi Province. This strain exhibited robust acclimation in high Pb^2+^ concentration cultures (2,000 mg/L and 1,500 mg/L), with peak values ranging from 2.92 cfu·mL^−1^ to 3.14 cfu·mL^−1^. Additionally, it demonstrated strong viability and adaptability in Cd^2+^ cultures at varying concentration gradients, showing growth even at concentrations exceeding 100 mg/L, with growth ranging from 1.45 cfu·mL^−1^ to 2.77 cfu·mL^−1^. Both *R. pseudoacacia* and *S. xanthantha* are suitable for cultivation in heavy metal-contaminated soils, as they can absorb and translocate heavy metals, such as Pb and Cd, resulting in an improved soil microenvironment. Combined with crop-applied WZ14 inoculants, the remediation efficacy was enhanced, resulting in increased soil AN and OC levels and decreased total Pb and active heavy metals compared to monoculture. Furthermore, the application of WZ14 significantly facilitated the uptake of Cd by *S. xanthantha*, particularly in the roots, where the Cd uptake reached 147.44 mg/kg after microbial inoculant. This represented a substantial increase of 130.79% compared to monoculture, signifying the root’s significant role in the remediation of soil Cd contamination by *S. xanthantha*. The community structure of soil microorganisms in heavy metal-contaminated areas exhibited significant differences between planting and combined remediation treatments, resulting in notable changes in microbial abundance and diversity. The combination of *R. pseudoacacia* monoculture with WZ14-*R. pseudoacacia* proved to be particularly effective in enhancing microbial diversity, as the WZ14-*R. pseudoacacia* soils displayed the highest abundance and diversity of microorganisms compared to other treatments. In heavy metal-contaminated soils, the dominant bacterial phyla were identified as *Proteobacteria*, *Bacteroidetes*, *Patescibacteria*, *Chloroflexi*, and *Acidobacteria*. The inoculation with WZ14 significantly increased the relative abundance of *Proteobacteria* and *Firmicutes*. Soil microorganisms have been proven crucial in remediating heavy metal-contaminated soils, with combined mycorrhizal agents and plant remediation proving highly promising for reducing heavy metal content and enhancing the soil environment.

## Data availability statement

The datasets presented in this study can be found in online repositories. The names of the repository/repositories and accession number(s) can be found at: NCBI—OR492361, PRJNA1012100.

## Author contributions

KZ: Conceptualization, Data curation, Writing – original draft. ZL: Investigation, Software, Writing – review & editing. CL: Formal Analysis, Methodology, Supervision, Writing – review & editing. JL: Methodology, Supervision, Writing – review & editing. JZ: Funding acquisition, Project administration, Resources, Writing – review & editing.
